# Will Temperature Effects or Phenotypic Plasticity Determine the Thermal Response of a Heterothermic Tropical Bat to Climate Change?

**DOI:** 10.1371/journal.pone.0040278

**Published:** 2012-07-03

**Authors:** Clare Stawski, Fritz Geiser

**Affiliations:** Centre for Behavioural and Physiological Ecology, Zoology, University of New England, Armidale, New South Wales, Australia; University of Sydney, Australia

## Abstract

The proportion of organisms exposed to warm conditions is predicted to increase during global warming. To better understand how bats might respond to climate change, we aimed to obtain the first data on how use of torpor, a crucial survival strategy of small bats, is affected by temperature in the tropics. Over two mild winters, tropical free-ranging bats (*Nyctophilus bifax*, 10 g, n = 13) used torpor on 95% of study days and were torpid for 33.5±18.8% of 113 days measured. Torpor duration was temperature-dependent and an increase in ambient temperature by the predicted 2°C for the 21^st^ century would decrease the time in torpor to 21.8%. However, comparisons among *Nyctophilus* populations show that regional phenotypic plasticity attenuates temperature effects on torpor patterns. Our data suggest that heterothermy is important for energy budgeting of bats even under warm conditions and that flexible torpor use will enhance bats’ chance of survival during climate change.

## Introduction

It is predicted that global warming will expose organisms to new thermal challenges and will result in poleward or altitudinal shifts of animals [1]. While a change in distribution to deal with climate change may be an option for some species, the response of animals is often too slow and not all can move, resulting in mismatching phenologies with potentially detrimental effects [2]–[3]. However, predictions on how animals might respond to climate change often rely on geographic ranges of species and the climate within these [4] and generally assume that species are static and have limited functional flexibility. Contrary to this, endothermic mammals, which have received little attention with regard to climate change [5], may adjust form and function to better suit the thermal conditions they were exposed to during their development [6]–[7]. This is especially true for heterothermic mammals capable of expressing torpor, which are known to be highly flexible in adjusting their energy requirements seasonally and regionally [8]–[13]. Importantly, the phenotypic plasticity of energy expenditure afforded by the opportunistic use of torpor appears to be a key factor in reducing the risk of extinction in mammals [14]–[15] and may be crucial in dealing with climate change and other anthropogenic disturbances.

Heterothermic endotherms use reductions in metabolic rate (MR) and body temperature (T_b_) during periods of torpor for energy conservation [16]. Torpor is used by diverse birds and mammals, often when food is limited, but also without apparent energetic stress to enhance fat stores for future energy demanding events, or to avoid predators [17]–[20]. Heterothermy is used by members of more than half of all mammalian orders [14] and is expressed especially in small species because their thermoregulatory energy expenditure can become costly during exposure to low ambient temperatures (T_a_).

Torpor use appears paramount in small temperate bats and it is well established that they often express a sequence of multiday torpor bouts (i.e. hibernation) during winter and short bouts of torpor lasting for part of the day in summer [21]–[23]. In contrast, it was believed in the past that bats inhabiting tropical regions do not use torpor at all because of mild environmental conditions [24]. This view is no longer supported because short bouts of torpor have been observed in captive tropical bats [25]–[27], and because subtropical bats express multiday torpor in the wild [28]–[30]. However, in the tropics essentially all information on the use of mammalian torpor in nature is currently limited to dwarf lemurs and tenrecs [31]–[34], despite the enormous diversity of tropical bats. Although bats comprise >20% of all mammals and the vast majority of these live in the tropics [27], only two individuals of a single species have been examined with regard to torpor in the wild [11].

Global warming is predicted to increase the numbers of bats exposed to tropical or at least warm conditions. Because this will affect energy use and foraging requirements, understanding the thermal biology of tropical bats in the wild will provide potential insights into how bats from other climates might respond to climate change. Although an increased T_a_ will reduce energy expenditure for normothermic thermoregulation at high T_b_, if bats do not use torpor at all their energy requirements will be substantially increased even under warm conditions [35]. The purpose of our study was twofold. We (i) aimed to provide the first long-term quantitative data on torpor use and activity patterns in relation to ambient conditions by tropical free-ranging northern long-eared bats, *Nyctophilus bifax*, that are entirely restricted to subtropical/tropical regions. We (ii) used these data and data from the literature to make predictions about how thermal energetics and torpor patterns of bats from tropical and other climate zones may be affected by climate change.

## Materials and Methods

Permits to undertake the research were provided by the UNE Animal Ethics Committee (AEC08/046, AEC09/058) and Queensland Parks and Wildlife Service (WITK04955708). A small subset of the data were published previously [36], however, a substantial amount of new data were added and all were re-analysed.

The field study was undertaken over two consecutive austral winters in June 2008 and July/August 2009 at Djiru National Park (17°50′S, 146°03′E), located in the tropical north of the Australian east coast and within the northern parts of the distribution range of *N. bifax* [37]. During both years, T_a_ was measured with temperature data loggers (±0.5°C, iButton thermochron DS1921G, Maxim Integrated Products, Inc., USA) in the shade 2 m above the ground. Thermal conditions during the two winters were similar: the overall mean T_a_ was 18.8±1.6°C and the mean T_a_ minima and maxima were 16.4±2.4°C and 21.9±1.7°C, respectively. The lowest and highest T_a_ recorded was 10.6 and 25.3°C, respectively.

Bats were netted for several hours after sunset. Captured bats were weighed to the nearest 0.1 g using an electronic scale and kept overnight. Captive bats were hand fed with mealworms and given water. On the following afternoon a small patch of fur from between the shoulder blades was removed and a temperature-sensitive radio-transmitter (∼0.5 g, LB-2NT, Holohil Systems Inc., Canada) was glued to the exposed skin using a latex adhesive (SkinBond, Smith and Nephew United, Australia). The pulse rate of these transmitters is temperature-dependent and all transmitters were calibrated to the nearest 0.1°C in a water bath between 5 and 40°C against a precision thermometer before attachment. External transmitters provide a reasonable measure of core T_b_ as T_skin_ of resting or torpid small mammals differs by <2°C from core T_b_ [38]. Transmitters worn and shed by bats (3 in 2008; 1 in 2009) were retrieved and re-calibrated 21 to 26 days after the initial calibration and were within 0.5°C of the initial calibration over the entire temperature range.

Bats were released at their capture site and on the following morning and on every day bats retained the transmitter each individual, identified by the frequency of its transmitter, was radio-tracked to its roost location. To automatically record T_skin_ every 10 min, remote receiver/loggers with antennae [39] were placed within range of the bats’ transmitter signal. Receiver/loggers were checked every morning when bats were located to ensure transmitter reception. Manual readings of the transmitter signals were taken daily to certify the accuracy of receiver/logger readings. Data from receiver/loggers were downloaded and batteries replaced every three days.

Data were obtained for a total of 35 bat days (*n* = 7 individuals, 4 females, 3 males; body mass: 10.4±0.7 g) in June 2008. During July/August 2009 data were obtained for a total of 78 bat days (*n* = 6 individuals, 4 females, 2 males; body mass: 9.9±0.7 g,). Mean body mass did not differ between years (P = 0.3, T = 1.1).

Torpor bouts are often defined as periods with T_b_ <30°C [40]. As the T_b_-T_skin_ differential during torpor is generally <2°C, we defined torpor bouts as the time when T_skin_ was <28°C. Data analyses were performed using StatistiXL (V 1.8, 2007); data are reported as means ± SD (*n*  =  number of individuals, *N*  =  number of observations). Means of each individual were used to calculate means for repeated measures. Results were considered significant when alpha was <0.05. To determine whether timing of arousals and torpor entries differed significantly from random, a Rayleigh test was used. T-tests were used to compare independent means; data of the sexes were pooled because they were statistically indistinguishable. Linear regressions were fitted by the least squares method and ANCOVAs were used to compare linear regressions. If no difference in slope between individuals or study periods was observed, data were pooled and regressed together.

## Results

### Torpor Patterns

**Figure 1:**
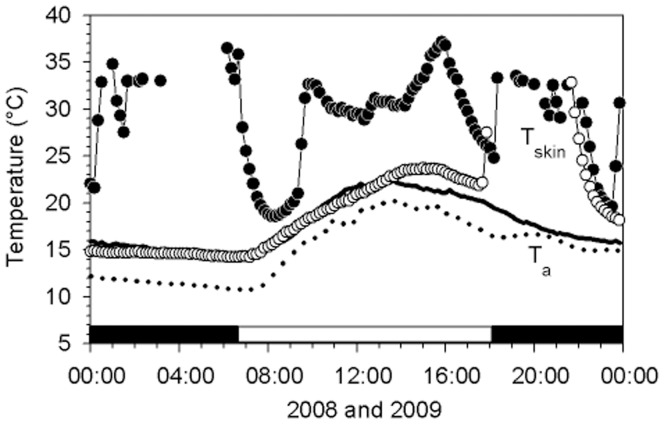
T_skin_ of two different individual *N. bifax* and T_a_ showing different patterns of torpor. The patterns shown are (i, T_skin_: open circles, T_a_: dotted line) an individual that remained torpid during the whole day and aroused only in the evening to possibly forage, and the second pattern shows (ii, T_skin_: closed circles, T_a_: smooth line) an individual displaying the typical morning and afternoon bouts of torpor along with an additional torpor bout during the night. The horizontal black and white bars at the bottom of the graphs represent night and day, respectively.

A total of 210 torpor bouts were recorded over both winters. Torpor was used on 83% (June 2008) and 100% (July/August 2009; both years combined 95%) of days on which data were collected. In both years, bats expressed different patterns of thermoregulation, entering 0 to 4 torpor bouts/day; some bats remained torpid for an entire day (5.7% of torpor days; Fig. 1). The two most common temporal patterns were one torpor bout/day (31.1%) and two torpor bouts/day (33.0%), typically with one bout in the morning and the other in the afternoon. Four bouts/day were rare (6.6%), but three bouts/day were relatively common (23.6%; Fig. 1), with the third bout occurring during the night before a possible early morning foraging period.

**Figure 2:**
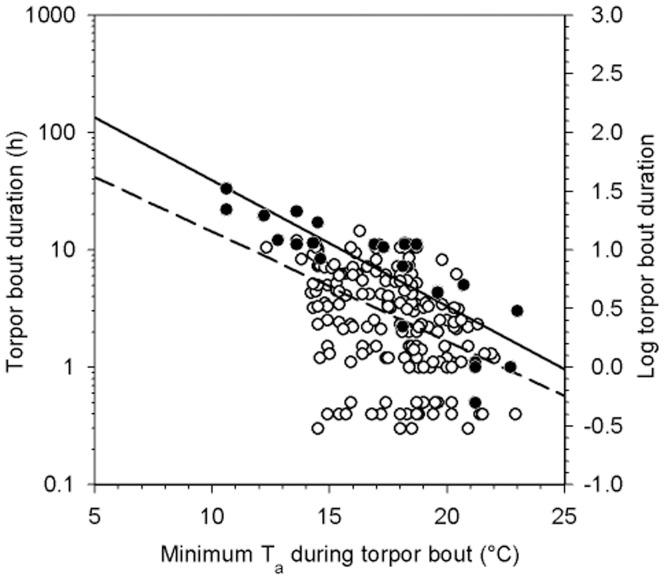
Duration of torpor bouts (log_10_) as a function of the minimum Ta of each torpor bout. All torpor bouts recorded are represented by the open circles and dashed line (log_10_ TBD = 2.1–0.09[T_a_ °C]; R^2^ = 0.2, P<0.001, F_1,209_ = 55.0). The two longest bouts recorded for each individual are represented by the closed circles and solid line (log_10_ TBD = 2.7 - 0.1[T_a_ °C]; R^2^ = 0.8, P<0.001, F_1,25_ = 73.3).

Mean torpor bout duration for both winters was 4.5±3.1 h (*n* = 13, *N* = 210; years did not differ: P = 0.7, T_11_ = 0.4). The longest torpor bout recorded was 33.3 h and a total of 31 torpor bouts (out of 210) were >10 h. Torpor bouts were negatively correlated with minimum T_a_ (R^2^ = 0.2, P<0.001; Fig. 2). The two longest torpor bouts recorded for each individual were strongly affected by minimum T_a_ (R^2^ = 0.8, P<0.001; Fig. 2) and the thermal response for this relationship was pronounced (Q_10_ = 10).

### Skin Temperature

**Figure 3:**
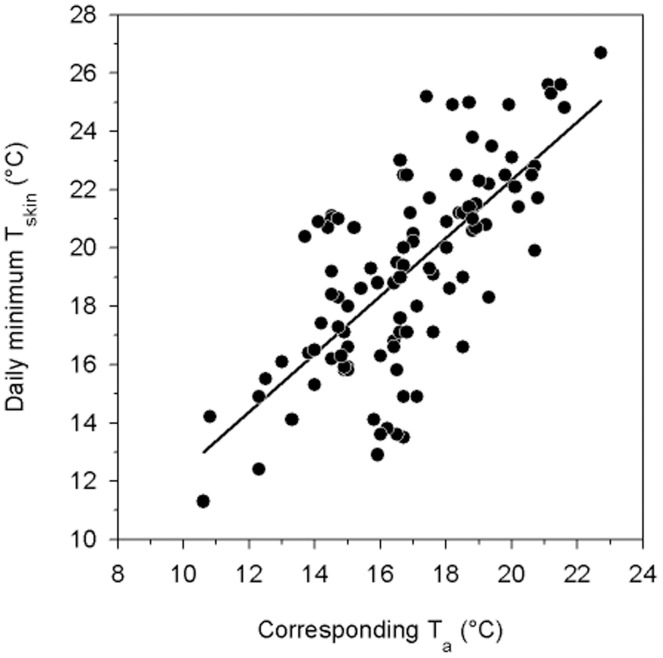
Daily torpid minimum T_skin_ of *N. bifax* as a function of T_a_ during winter. This relationship is represented by the following equation: minimum T_skin_(°C) = 2.4+1.0[T_a_°C]; R^2^ = 0.5, P<0.001, F_1,100_ = 101.2.

Mean daily minimum T_skin_ in torpid *N. bifax* during both winters was 20.1±3.1°C (*n* = 13, *N* = 102; years did not differ: P = 0.1, T_11_ = 1.8). The lowest individual T_skin_ value recorded was 11.3°C (T_a_ = 10.6°C). The daily minimum torpid T_skin_ was correlated with T_a_ (R^2^ = 0.5, P<0.001; Fig. 3). The mean differential between daily minimum T_skin_ during torpor and the corresponding T_a_ was 2.1±1.7°C (*n* = 13, *N* = 101; years did not differ: P = 0.7, T_6_ = 0.4).

### Timing of Torpor and Activity

**Figure 4:**
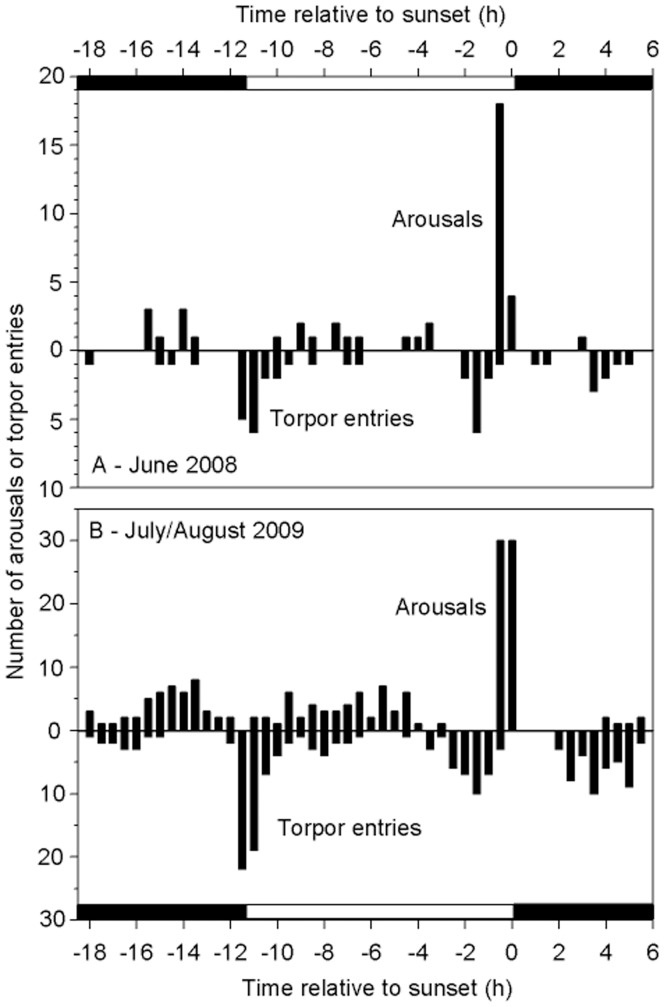
Timing of activity and torpor. Distribution of times of arousals from torpor (top half of graphs) and entries into torpor (bottom half of graphs) of *N. bifax* during (A) June 2008 and (B) July/August 2009 relative to the time of sunset (0 hours). Each individual contributed several points to these graphs, ranging from 2 to 41 data points. Each bar represents a 30 minute period. The horizontal black and white bars at the top and bottom of the graphs represent night and day, respectively.

**Figure 5:**
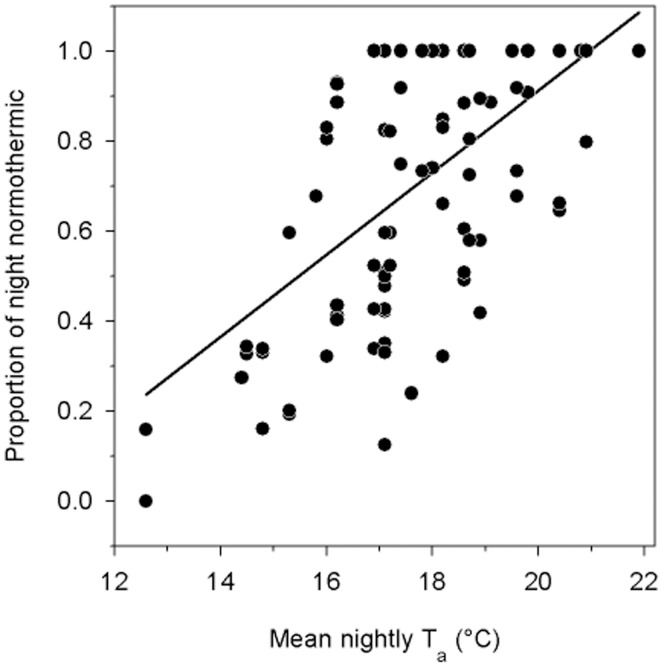
The proportion of a night that *N. bifax* spent normothermic as a function of mean nightly T_a_. This relationship is represented by the following equation: proportion night normothermic  =  −0.9+0.09[T_a_ °C]; R^2^ = 0.4, P<0.001, F_1,95_ = 51.1.

Entries into torpor in 2008 (Fig. 4) displayed a peak at a mean time (angle) of 8∶14±5∶10 h (*n* = 7, *N* = 43); in 2009 the mean time was 2∶50±5∶15 h (*n* = 6, *N* = 167; 2009), but timing of torpor entries did not differ significantly from random (2008: Rayleigh Z = 0.3, P = 0.2; 2009: Z = 0.5, P = 0.2). Arousals were non-randomly distributed in 2008 (Z = 6.0, P = 0.002) with a mean time of 16∶20±4∶17 h (*n* = 7, *N* = 43), but not in 2009 (mean: 15∶02±5∶04 h, *n* = 6, *N* = 167, Z = 2.5, P = 0.1). Evening arousals likely for foraging occurred at sunset ±00∶06 h (*n* = 7, *N* = 22; 2008) and slightly before sunset 00∶06±00∶04 h (*n* = 6, *N* = 60; 2009). The proportion of a night *N. bifax* remained normothermic during both winters was positively correlated with mean nightly T_a_ (R^2^ = 0.4, P<0.001; Fig. 5).

## Discussion

Our study provides the first long-term quantitative data of torpor use and patterns in a tropical bat in its natural environment. It also is the first to show that tropical bats can remain torpid for >1 day. While this was a rare occurrence, torpor was frequently used (95% of all study days) even though weather conditions were mild. Our extensive field study of *N. bifax* and a recent brief field study on two individual *N. geoffroyi* during winter in a tropical habitat [11] confirm earlier findings from laboratory work [25]–[26] that torpor is indeed widely used by tropical bats for energy conservation in the wild. Further, data on tropical bats and on other mammals such as lemurs and tenrecs from Madagascar [31]–[33] show that, contrary to the widely held view, torpor use is prevalent in tropical regions. Frequent use of torpor by bats during winter in tropical regions, as reported here for *N. bifax*, highlights the importance of energy conservation for small microbats even under relatively mild conditions.

Several different patterns of torpor were expressed by *N. bifax* during both winters, with a peak in arousals from torpor bouts just before sunset. The variation in use of torpor by *N. bifax* is likely in response to variations in weather conditions and food abundance and *N. bifax* were normothermic/active longer on warmer nights like other bat species [23], [30]. For insectivorous bats specifically it makes sense to use more torpor at low T_a_ to save energy when feeding is difficult. In the current study torpor bout duration at minimum T_a_s >14°C varied widely above and below the regression line, whereas at minimum T_a_s <14°C all torpor bouts fell above the regression line (Fig. 2). This effect of T_a_ may reflect insect availability, which decreased significantly at T_a_s <16°C in the study region [41]. It is also important to note in this context that the thermal response of torpor bout duration of *N. bifax* was pronounced (Q_10_ = 10), which is about 3-fold of that usually observed in temperate bats (Q_10_ = 2.6 to 3.9, [42]). This high thermal sensitivity will permit tropical *N. bifax* to use relatively long torpor bouts in response to a small reduction of T_a_ and, on the other hand, be active for much of the night when T_a_ increases.

The T_skin_ of torpid *N. bifax* approached T_a_, with a minimum T_a_-T_skin_ differential of ∼2°C. Even on particularly cold days when T_skin_ was very low, bats apparently continued to thermo-conform because the T_a_-T_skin_ differential remained constant suggesting that torpid bats did not thermoregulate. The lowest T_skin_ recorded was 11.3°C, which is rather low for a tropical mammal and suggests that individuals of this population of *N. bifax* can approximate the low T_b_s that are characteristic of hibernation in cold climates [16], [35]. This is supported by laboratory data showing that tropical *N. bifax* commenced to thermoregulate during torpor only at T_a_ 6.7°C and the minimum T_b_ was 7.3°C [43]. Therefore, the generally high T_skin_ in the current study compared to cold-climate hibernators appears to be mainly a reflection of the high T_a_s bats experienced. However, the minimum T_b_ measured in the laboratory was also somewhat higher than in temperate hibernators and this trait appears to be selected by the T_a_ animals are exposed to in the wild [8], [36].

What are the implications of our data for the effect of climate change on bats? We used two approaches to assess this: (i) We assumed that the thermal physiology of bats is constant and estimated using data from the present study and published data [43] how a predicted T_a_ increase by 2°C will affect torpor patterns and consequently energy use, and (ii) used data on thermal biology from free-ranging subtropical and temperate *Nyctophilus* populations to test these predictions.

If we (i) use data presented here and those on thermal energetics of tropical *N. bifax* [43], we can estimate energy expenditure during torpor from mean T_skin_ and MR regressions because the animals were thermo-conforming and rewarmed largely passively, in comparison to normothermic thermoregulation over the same time period. At a mean T_a_ of 18.8°C, *N. bifax* remained torpid for 33.5% of the time, or 8.02 h/day, with a mean T_skin_ of 24.3°C during torpor using 525 J (assuming 19.7 kJ/lO_2_ for metabolised fat, [44]). Resting normothermic bats at T_a_ 18.8°C would have used 7,710 J, and the energy saved by using torpor would be 7,185 J (895.8 J/h) or 28% of the daily energy expenditure of a 10-g temperate bat (25.88 kJ/d, [45]). The thermal response of torpor bout duration (Fig. 2) predicts that a 2°C increase in T_a_ will shorten the duration of torpor to 21.8% of the time (5.23 h/day), and energy expenditure during torpor will be 467 J. Resting normothermic bats at T_a_ 20.8°C would need less energy for thermoregulation (4,131 J) and energy savings due to torpor would decrease to 3,664 J (700 J/h) or 14% of the predicted daily energy expenditure [45]. Thus, even at the higher T_a_, energy savings by using torpor are substantial and biologically meaningful.

**Figure 6:**
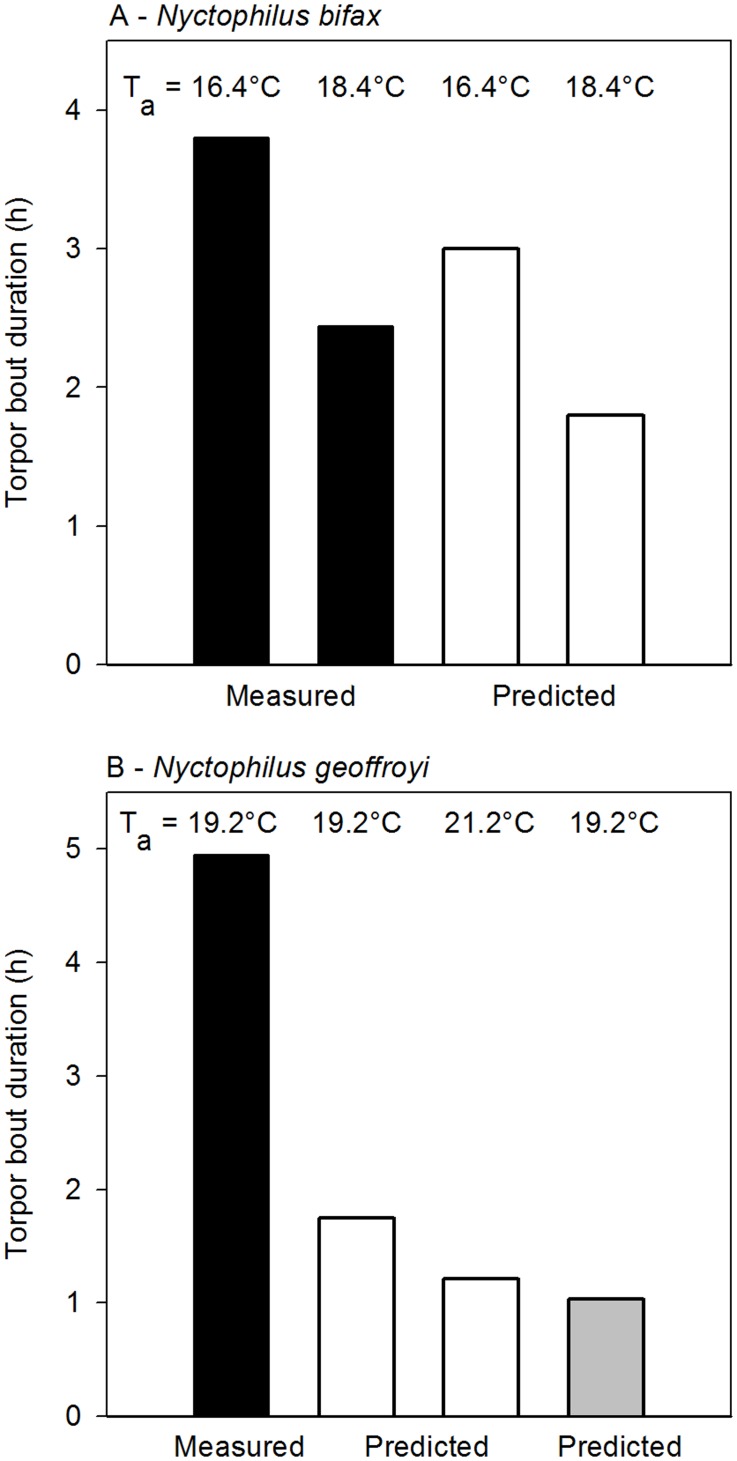
Measured and predicted changes in torpor bout duration in relation to predicted increases in T_a_. Measured (black bars) and predicted (white and grey bars) torpor bout duration in tropical and subtropical *N. bifax* (A), and in tropical and temperate *N. geoffroyi* (B). Measured values are those obtained at the tropical sites at the mean minimum T_a_ (T_a_ 16.4°C and T_a_ 16.4+2°C *N. bifax*, T_a_ 19.2°C only for *N. geoffroyi* because no T_a_-torpor bout duration regression is available). Predicted torpor bout durations were calculated from regressions in subtropical *N. bifax* [36] and from temperate *N. geoffroyi* in summer (white bars, [22]) and winter (grey bar, [23]).

Pronounced discrepancies were observed when we (ii) examined whether and how the thermal biology of populations of bat species in the wild differs from that predicted from regressions. In *N. bifax* mean torpor bout duration of a subtropical population [36] is predicted to decrease from 3.0 to 1.8 h if T_a_ increases by 2°C from the tropical mean minimum T_a_ of 16.4°C to 18.4°C (Fig. 2, 6). However, measured torpor bout duration at the tropical site at T_a_ 16.4°C is in fact 3.8 h (127% of predicted) and 2.4 h at T_a_ 18.4°C (136% of predicted). This shows that temperature effects on torpor bout duration vary among populations and suggests that either the tropical bats have acclimated or have been selected to maintain relatively long torpor bouts at warm T_a_. Measured and predicted values differ even more in the congener *N. geoffroyi* (Fig. 6), distributed over almost the entire Australian continent. Data from *N. geoffroyi* from a temperate region in summer [22] predict that torpor bout duration at the mean minimum T_a_ of 19.2°C in tropical Northern Territory is only 1.7 h and will decline to 1.2 h with a 2°C rise of T_a_. Measured torpor bout duration in tropical *N. geoffroyi* at a mean minimum T_a_ of 19.2°C is in fact 4.9 h [11], 2.8-times that predicted from temperate bats. Winter data [23] predict that torpor bout duration of temperate *N. geoffroyi* at a minimum T_a_ of 19.2°C and a mean T_skin_ of 26.2°C is only 1.0 h, only 21% of that measured in the tropics.

As torpor is usually associated with cold, whereas climate change with global warming, what do our projections actually tell us about bats in a warming climate? During periods of high temperatures, heat waves are known to induce hyperthermia and can kill large pteropodid bats [46]. However, pteropodids comprise only a rather small number (∼20%) of bat species and many large members of this family may roost at exposed sites often directly affected by T_a_ extremes. In contrast, most ‘microbats’ roost in sheltered areas like caves, mines, houses, under bark or leaves that are buffered from thermal extremes, and, in addition to using torpor, can also be tolerant of extremely high T_a_ exceeding 50°C [47]. Thus, our and previously available data suggest that by using torpor opportunistically and by being able to tolerate high T_a_, small bats may be better equipped to deal with climate change than is predicted from bio-climatic data, especially those species that can shift their distribution to cooler habitats [48].

Obviously, there will be a limit to how far T_a_ can rise before torpor will become ineffective and a tolerance of high T_a_ will be exceeded. Moreover, some hibernating mammals are restricted to mountain tops that do not permit further altitudinal adjustments to climate change [2], [49]. Consequently, those heterothermic mammals with a period of winter dormancy that is strongly dependent on historical phenological patterns, which are also often those restricted to limited mountain habitats, are likely to be adversely affected. Recent evidence also shows that hibernating bats are susceptible to new pathogens, such as white-nose syndrome, which kills bats by interfering with their seasonal hibernation [50]. In contrast, opportunistic heterothermic species and those able to use torpor efficiently even under varying thermal conditions, may be able to deal with climate change and other detrimental factors better than predictions from current models might suggest.
